# Supplementary material for: Asymmetric magnetization switching and programmable complete Boolean logic enabled by long-range intralayer Dzyaloshinskii-Moriya interaction
Source: Nat Commun. 2024 Apr 6;15:2978. doi: 10.1038/s41467-024-47375-5 (PMC10998899; doi:10.1038/s41467-024-47375-5)
Supplement: Supplementary file 1 — Supplementary Information [file 41467_2024_47375_MOESM1_ESM.pdf]

Supplementary Information for

Asymmetric magnetization switching and programmable complete Boolean logic enabled by long-range intralayer Dzyaloshinskii-Moriya interaction

Qianbiao Liu,[1,2] Long Liu,[3] Guozhong Xing,[3] Lijun Zhu[1,2]*

[1]State Key Laboratory for Superlattices and Microstructures, Institute of Semiconductors, Chinese Academy of Sciences, Beijing 100083, China

[2]College of Materials Science and Opto-Electronic Technology, University of Chinese Academy of Sciences, Beijing 100049, China

[3]Institute of Microelectronics, Chinese Academy of Sciences, Beijing 100029, China

Contents

Supplementary Table 1. Magnetic properties of the samples.

Supplementary Figure 1. Sharp interface.

Supplementary Figure 2. Field and current switching data for Cr 5/Ti 1/FeCoB 1, Ta 5/FeCoB 1, Ir 5/Co1, and Pd 5/Co 1.

Supplementary Figure 3. Kerr microscopy image for the Ir 5.4/FeCoB 1.

Supplementary Note 1. Nonzero total DMI.

Supplementary Table 1| Magnetic properties of the samples. For data in this work, the saturation magnetization (M_s) is estimated from SQUID measurement, the perpendicular magnetic anisotropy field (H_k) is estimated from the parabolic dependence of the first harmonic Hall voltage ($V_{1\omega}$) on the in-plane bias field in the small field range, i.e., to $V_{1\omega}= V_{AH}\,(1-H_x^2/2H_k^2)$, with V_{AH} being the anomalous Hall voltage. the interfacial perpendicular magnetic anisotropy energy density (K_s) is estimated following the relation of $H_k \approx -4\pi M_s+2K_s/M_s t$ with t being the layer thickness of the magnetic layer. The width of the domain wall (Δ) and its upper limit (Δ_{upper}) are estimated using Equation (6) and the simplified relation $\Delta_{upper} \approx \sqrt{2A/H_k M_s}$, respectively, where A is the exchange stiffness of the magnetic layer.

Samples	This work data					Reference data			
	M_s (emu/cm^3)	H_k (kOe)	K_s (erg/cm^2)	Δ (nm)	Δ_{upper} (nm)	M_s (emu/cm^3)	K_s (erg/cm^2)	D_s (10^{-7} erg/cm)	Ref.
Pt/Co	1370	7.0	1.66	3.7	5.6	1273	1.3	1.2	38
Ir/Co	1400	5.4	1.61	4.0	6.3	1400	>1.13	0.34	49
Pd/Co	1400	10.7	1.98	3.1	4.5	1500	1.79	0.01	54
Cr/Ti/FeCoB	1215	2.8	1.09	5.0	7.0	1000	0.68	0.06	53
Ir/FeCoB	1200	6.7	1.31	3.7	4.6	956	0.38	0.21	49
Ta/FeCoB	1185	1.5	0.97	6.1	9.7	1200	0.95	0.22	52
W/FeCoB	1180	4.0	1.11	4.5	6.0	1200	0.92	0.38	51

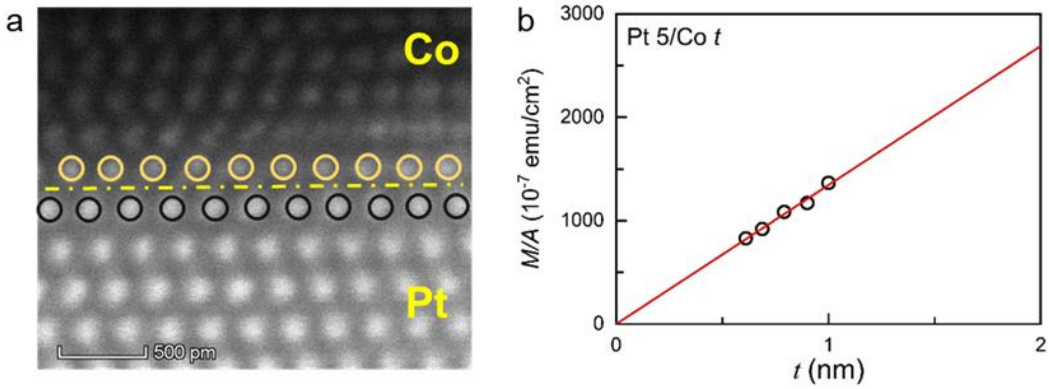

Supplementary Fig. 1. **Sharp interface. a.** Scanning transmission electron microscopy image of a Pt/Co interface with the heavy Pt atoms brighter than the light Co atoms, indicating the reasonably sharp interface. **b.** Thickness dependence of magnetic moment per area for a Pt/Co/MgO sample. The solid line represents the best linear fit. The negligibly small intercept of the linear fit suggests the absence of any significant magnetic dead layer at the Pt/Co or Co/MgO interfaces.

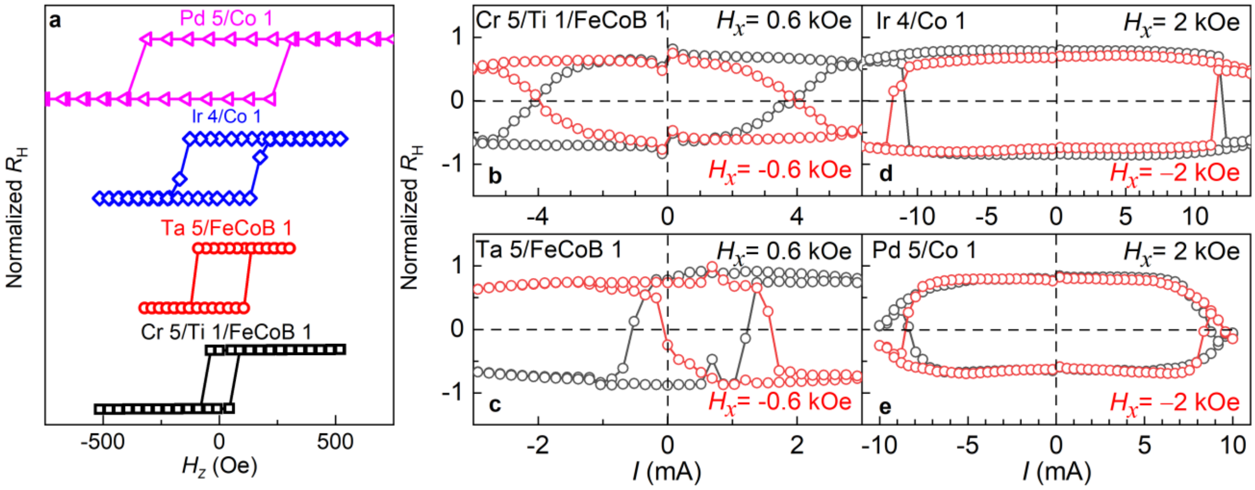

Supplementary Fig. 2| Field and current switching data for Cr 5/Ti 1/FeCoB 1, Ta 5/FeCoB 1, Ir 5/Co1, and Pd 5/Co 1. (**a**) The anomalous Hall resistance R_H vs perpendicular magnetic field H_z. (**b-e**) R_H vs dc current inside the HM layer (I), with the applied in-plane field H_x of ± 0.6 kOe for the FeCoB samples and ± 2 kOe for the Co samples. The lines are to guide the eyes.

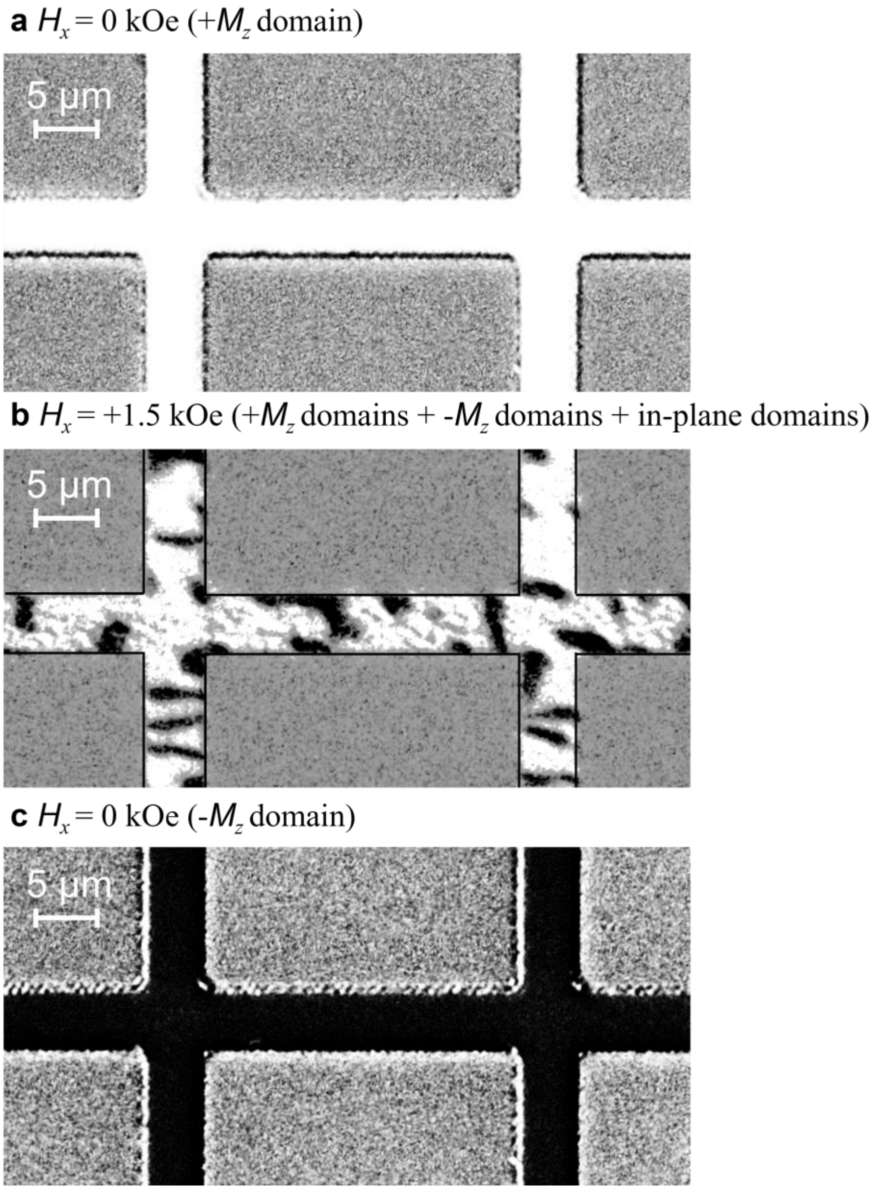

Supplementary Fig. 3| Kerr microscopy image for the Ir 5.4/FeCoB 1. (**a**) +M_z domain (white) at remanence state after saturation by a positive magnetic field H_z. (**b)** Coexistence of +M_z domains (white), -M_z domain (black), and in-plane domains (gray) during switching. (**c**) -M_z domain (black) after full switching.

Supplementary Note 1| Nonzero total DMI.

The nonzero total DMI effects in practical samples are proved by their consequences (e.g., the interfacial DMI-induced frequency difference between counterpropagating Damon-Eshbach spin waves, the interlayer or intralayer DMI-induced switching asymmetry). As shown in the Supplementary Fig. 4a-c, the various DMI effects, including the interfacial DMI, interlayer DMI, and intralayer DMI, appear to cancel out for the center spins (or domains) when the DMI vectors are of opposite signs on the left and right sides (1 and 2 in the Supplementary Fig.4a-c). This likely suggests that the total DMI effects of practical samples are mainly from the spins (domains) and heavy metal atoms that are in microscopic configurations more complex than those in Supplementary Fig. 4a-c.

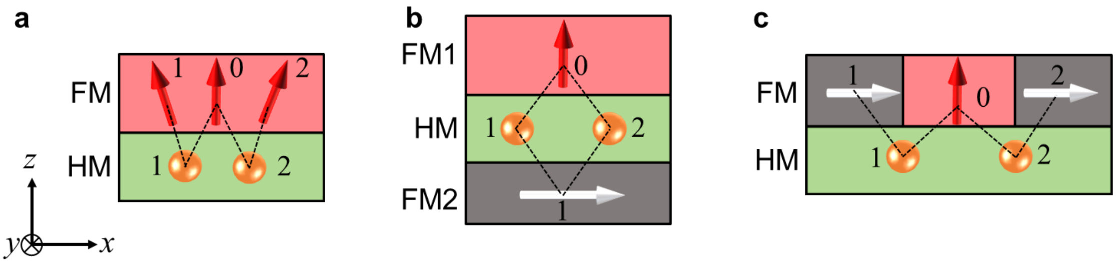

Supplementary Fig. 4| Total DMI. **a**, interfacial DMI, **b**, interlayer DMI, **c**, intralayer DMI. FM and HM are short for the ferromagnetic layer and the heavy metal, respectively.

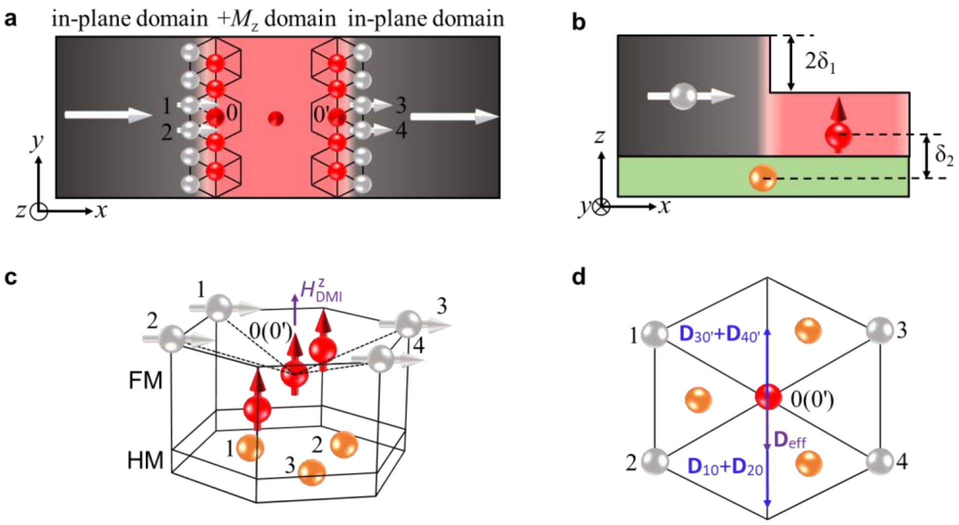

Supplementary Fig. 5| A circumstance for non-zero long-range perpendicular DMI field. **a**. Top-view schematic of in-plane domain/out-of-plane (+M_z) domain/in-plane domain. **b**. Side-view schematic indicating the thickness-induced anisotropy variation. **c**. Relative location of the nano-domains of the interlayer DMI. **d**. Top view of the interacting nano-domains.

Below we provide a toy model for the nonzero total long-range perpendicular DMI field in a heavy metal/ferromagnet (HM/FM) strip, in which the variation of the perpendicular magnetic anisotropy (PMA) is induced by the atomic thickness variation. When a sufficient in-plane magnetic field is applied along the *x* direction, the low PMA region will be aligned in-plane first as shown by the top-view schematic in the Supplementary Fig. 5a and the side-view schematic in the Supplementary Fig. 5b. Both the in-plane domains and the out-of-plane domains can be viewed as composed of small close-packed hexagonal moment nanodomains. The moments within a single big domain are ferromagnetically coupled, while the small edge-region out-of-plane moment nanodomains (red) are chiral-coupled to the neighboring in-plane moment nanodomains (gray). The latter, the nearest neighboring interaction, can be approximated as the dominant source of the total DMI field exerted on the out-of-plane domain region. Thus, when the left and right domain boundaries are considered at the same time, such DMI coupling can be estimated from the hexagonal unit cell in the Supplementary Fig. 5c. For the sake of convenience, we note the central out-of-plane domain as $FM_{0(0')}$, the outer in-plane domain as FM_n (n= 1, 2, 3, 4) and the heavy metal atoms as HM_m (m=1,2,3). According to the Levy-Fert three-point model, the DMI vector is

$$\mathbf{D}_{n0(0')} = -\zeta D \mathbf{r}_{mn} \times \mathbf{r}_{m0(0')}, \qquad (1)$$

where $\mathbf{r}_{mn}$ is the unit direction vector from the HM_m to the FM_n, and $\mathbf{r}_{m0(0')}$ is the unit direction vector from the HM_m to the $FM_{0(0')}$. Defining the position of $FM_{0(0')}$ as the origin of the coordinates, the domain wall width as Δ, the thickness difference between strong PMA and weak PMA domains as $2\delta_1$, the vertical distance between HM and the center of the out-of-plane domain $FM_{0(0')}$ as δ_2, we obtain the coordinates of the FM_n and HM_m as: $FM_{0(0')} = (0, 0, 0)$, $FM_1 = (-\frac{\sqrt{3}\Delta}{2}, \frac{\Delta}{2}, \delta_1)$, $FM_2 = (-\frac{\sqrt{3}\Delta}{2}, -\frac{\Delta}{2}, \delta_1)$, $FM_3 = (\frac{\sqrt{3}\Delta}{2}, \frac{\Delta}{2}, \delta_1)$, $FM_4 = (\frac{\sqrt{3}\Delta}{2}, -\frac{\Delta}{2}, \delta_1)$, $HM_1 = (-\frac{\Delta}{\sqrt{3}}, 0, -\delta_2)$, $HM_2 = (\frac{\Delta}{2\sqrt{3}}, \frac{\Delta}{2}, -\delta_2)$, $HM_3 = (\frac{\Delta}{2\sqrt{3}}, -\frac{\Delta}{2}, -\delta_2)$.

Accordingly, the unit direction vectors and the DMI vectors are

$$\mathbf{r}_{11} = \left(-\frac{\Delta}{2\sqrt{\Delta^2+3(\delta_1+\delta_2)^2}}, \frac{\sqrt{3}\Delta}{2\sqrt{\Delta^2+3(\delta_1+\delta_2)^2}}, \frac{2\sqrt{3}\delta_1}{\sqrt{\Delta^2+3(\delta_1+\delta_2)^2}}\right), \quad (2)$$

$$\mathbf{r}_{10} = \left(\frac{\Delta}{\sqrt{\Delta^2+3\delta_2^2}}, 0, \frac{\sqrt{3}\delta_2}{\sqrt{\Delta^2+3\delta_2^2}}\right), \quad (3)$$

$$\mathbf{D}_{10} = -D\mathbf{r}_{11}\times\mathbf{r}_{10} = D\left(-\frac{3\Delta\delta_2}{2\sqrt{[\Delta^2+3(\delta_1+\delta_2)^2](\Delta^2+3\delta_2^2)}}, -\frac{4\sqrt{3}\Delta\delta_1+\sqrt{3}\Delta\delta_2}{2\sqrt{[\Delta^2+3(\delta_1+\delta_2)^2](\Delta^2+3\delta_2^2)}}, \frac{\sqrt{3}\Delta^2}{2\sqrt{[\Delta^2+3(\delta_1+\delta_2)^2](\Delta^2+3\delta_2^2)}}\right), \quad (4)$$

$$\mathbf{r}_{12} = \left(-\frac{\Delta}{2\sqrt{\Delta^2+3(\delta_1+\delta_2)^2}}, -\frac{\sqrt{3}\Delta}{2\sqrt{\Delta^2+3(\delta_1+\delta_2)^2}}, \frac{2\sqrt{3}\delta_1}{\sqrt{\Delta^2+3(\delta_1+\delta_2)^2}}\right), \quad (5)$$

$$\mathbf{r}_{10} = \left(\frac{\Delta}{\sqrt{\Delta^2+3\delta_2^2}}, 0, \frac{\sqrt{3}\delta_2}{\sqrt{\Delta^2+3\delta_2^2}}\right), \quad (6)$$

$$\mathbf{D}_{20} = -D\mathbf{r}_{12}\times\mathbf{r}_{10} = D\left(\frac{3\Delta\delta_2}{2\sqrt{[\Delta^2+3(\delta_1+\delta_2)^2](\Delta^2+3\delta_2^2)}}, -\frac{4\sqrt{3}\Delta\delta_1+\sqrt{3}\Delta\delta_2}{2\sqrt{[\Delta^2+3(\delta_1+\delta_2)^2](\Delta^2+3\delta_2^2)}}, -\frac{\sqrt{3}\Delta^2}{2\sqrt{[\Delta^2+3(\delta_1+\delta_2)^2](\Delta^2+3\delta_2^2)}}\right), \quad (7)$$

$$\mathbf{r}_{23} = \left(\frac{\Delta}{\sqrt{\Delta^2+3(\delta_1+\delta_2)^2}}, 0, \frac{2\sqrt{3}\delta_1}{\sqrt{\Delta^2+3(\delta_1+\delta_2)^2}}\right), \mathbf{r}_{20'} = \left(-\frac{\Delta}{2\sqrt{\Delta^2+3\delta_2^2}}, -\frac{\sqrt{3}\Delta}{2\sqrt{\Delta^2+3\delta_2^2}}, \frac{\sqrt{3}\delta_2}{\sqrt{\Delta^2+3\delta_2^2}}\right), \quad (8)$$

$$\mathbf{D}_{30'} = -D\mathbf{r}_{23}\times\mathbf{r}_{20'} = D\left(-\frac{3\Delta\delta_1}{\sqrt{[\Delta^2+3(\delta_1+\delta_2)^2](\Delta^2+3\delta_2^2)}}, \frac{\sqrt{3}\Delta\delta_1+\sqrt{3}\Delta\delta_2}{\sqrt{[\Delta^2+3(\delta_1+\delta_2)^2](\Delta^2+3\delta_2^2)}}, \frac{\sqrt{3}\Delta^2}{2\sqrt{[\Delta^2+3(\delta_1+\delta_2)^2](\Delta^2+3\delta_2^2)}}\right), \quad (9)$$

$$\mathbf{r}_{34} = \left(\frac{\Delta}{\sqrt{\Delta^2+3(\delta_1+\delta_2)^2}}, 0, \frac{2\sqrt{3}\delta_1}{\sqrt{\Delta^2+3(\delta_1+\delta_2)^2}}\right), \quad (10)$$

$$\mathbf{r}_{30'} = \left(-\frac{\Delta}{2\sqrt{\Delta^2+3\delta_2^2}}, \frac{\sqrt{3}\Delta}{2\sqrt{\Delta^2+3\delta_2^2}}, \frac{\sqrt{3}\delta_2}{\sqrt{\Delta^2+3\delta_2^2}}\right), \quad (11)$$

$$\mathbf{D}_{40'} = -D\mathbf{r}_{34}\times\mathbf{r}_{30'} = D\left(\frac{3\Delta\delta_1}{\sqrt{[\Delta^2+3(\delta_1+\delta_2)^2](\Delta^2+3\delta_2^2)}}, \frac{\sqrt{3}\Delta\delta_1+\sqrt{3}\Delta\delta_2}{\sqrt{[\Delta^2+3(\delta_1+\delta_2)^2](\Delta^2+3\delta_2^2)}}, -\frac{\sqrt{3}\Delta^2}{2\sqrt{[\Delta^2+3(\delta_1+\delta_2)^2](\Delta^2+3\delta_2^2)}}\right). \quad (12)$$

Thus, the total DMI vector ($\mathbf{D}_{eff}$) of the $FM_{0(0')}$ due to the FM_n (n = 1, 2, 3, 4) is

$$\mathbf{D}_{eff} = \mathbf{D}_{10} + \mathbf{D}_{20} + \mathbf{D}_{30'} + \mathbf{D}_{40'} = \left(0, \frac{(-2\sqrt{3}\delta_1+\sqrt{3}\delta_2)\Delta D}{\sqrt{[\Delta^2+3(\delta_1+\delta_2)^2](\Delta^2+3\delta_2^2)}}, 0\right), \quad (13)$$

$$|\mathbf{D}_{eff}| = \left|\frac{(-2\sqrt{3}\delta_1+\sqrt{3}\delta_2)\Delta D}{\sqrt{[\Delta^2+3(\delta_1+\delta_2)^2](\Delta^2+3\delta_2^2)}}\right|. \quad (14)$$

$\mathbf{D}_{eff}$ points to the y direction (Supplementary Fig. 5d) and is nonzero in magnitude when $\delta_2 \neq 2\delta_1$ as is usually the case for the HM/FM bilayers. The perpendicular DMI field exerted on the out-of-plane domains by the in-plane domains $\mathbf{M}_x$ is $H_{\mathrm{DMI}}^{z} = \mathbf{M}_x\times\mathbf{D}_{eff}$, pointing to the z direction.
